# Where is the exact origin of narrow premature ventricular contractions manifesting qR in inferior wall leads?

**DOI:** 10.1186/s12872-016-0240-4

**Published:** 2016-04-04

**Authors:** Cheng Zheng, Jin Li, Jia-Xuan Lin, Lu-Ping Wang, Jia-Feng Lin

**Affiliations:** Department of Cardiology, Second Affiliated Hospital of Wenzhou Medical University, 109 Xueyuan Road, Wenzhou, Zhejiang China

**Keywords:** Radiofrequency catheter ablation, Premature ventricular contractions, Left anterior fascicle, L-RCC ILT

## Abstract

**Background:**

In recent years, radiofrequency catheter ablation(RFCA) has been established as an effective therapy for idiopathic premature ventricular contractions (PVCs), however, its effect on the narrow PVCs (QRS duration < 130 msec) with qR pattern in inferior leads, may not been fully concluded.

**Methods:**

Characteristics of 12-lead electrocardiogram (ECG) and electrophysiologic recordings were analyzed in 40 patients with symptomatic PVCs manifesting narrow QRS complex with qR pattern in inferior leads. The procedure of RFCA was performed based on pace mapping and activation mapping.

**Results:**

Among the 40 patients with narrow PVCs, complete elimination of PVCs was achieved by RFCA in 35 patients during a median follow-up period of 23 months. Successful ablation was achieved on 19 patients at the sites where earliest Purkinje potentials were recorded in left ventricular anterosuperior septum, thus PVCs arising from left anterior fascicle (LAF) were confirmed, for these PVCs, the QRS morphology were right bundle branch and left posterior fascicle block (RBBB + LPFB) with rightward axis, the average QRS duration 116.07 ± 7.96 ms, R or rsR’in lead V1,with transition zone ahead of lead V1 in precordial leads. Another 16 successful RFCA were achieved by energy delivery at interleaflet triangle(ILT) between right coronary cusp(RCC) and left coronary cusp(LCC) where no Purkinje potentials were recorded, for narrow PVCs arising from the L-RCC ILT, the QRS morphology were similar to the PVCs arising from LAF but much narrower in QRS duration (100.44 ± 3.49 vs. 116.07 ± 7.96 ms, *p* < 0.05), they were also R or Rs in lead V1 with the transition zone ahead of lead V1. For 5 symptomatic narrow PVCs failed to the procedure of RFCA, their electrocardiographic characteristics showed that the narrowest QRS duration (91.80 ± 6.94 vs. 100.44 ± 3.49, 116.07 ± 7.96 ms, *p* < 0.05), rs or rS (r/s or r/S≦1) morphology in lead V1 with the precordial transition zone behind lead V3.

**Conclusions:**

Most of idiopathic PVCs of narrow QRS duration (<130 msec) with qR pattern in inferior leads can be cured by the procedure of RFCA. On the basis of our study, we proposed that for narrow PVCs presenting qR pattern in inferior leads, when the ablation procedure failed at proximity of LAF within left anterosuperior septum, mapping and ablation in L-RCC ILT can be tried. The present findings can be helpful for planning catheter ablation for narrow PVCs manifesting qR in inferior leads.

## Background

Idiopathic premature ventricular contractions(PVCs) are the most common arrhythmias observed in patients without structural heart disease [1]. Though the myocardium around the right ventricular outflow tract is a major source of idiopathic PVCs, still a minority of PVCs originating from left ventricular outflow tract, the vicinity of mitral and tricuspid valve, and the other structures of heart [2–7]. Catheter ablation is demonstrated to be a low-risk and often effective treatment to eliminate PVCs and associated symptoms [8–12]. Recent studies shows in patients with PVC-induced cardiomyopathy, cardiac function frequently restored after successful ablation. In addition, successful ablation can prevent patients from the side effects of long-term antiarrhythmias drugs. It has been already demonstrated that RFCA was also an effective therapy for idiopathic left anterior fascicular ventricular tachycardia, which presented the electrocardiographic characteristics of narrow QRS complex with qR pattern in inferior wall leads [13]. In the present study, a series of narrow PVCs with qR pattern in inferior leads, which were similar to left anterior fascicular tachycardia in QRS morphology, undergoing RFCA in our center, were retrospectively reviewed and classified into 3 groups based on RFCA results. Informations on the relationships between the origins of PVCs and their electrocardiographic and electrophysiological characteristics were concluded.

## Methods

### Study population

From September 2006 to June 2015, a total 895 patients without structural heart disease underwent catheter ablation for PVCs/IVTs in our centre. Forty consecutive patients (15 men and 25 women; age 33.53 ± 12.48 years) showing narrow PVCs (QRS duration < 130 msec) with qR pattern in at least one inferior leads were included in the present study. The baseline clinical characteristics of the 40 patients were shown in Table 1. The average duration of disease (from the time of first symptomatic onset to the ablation procedure) was 20.08 ± 11.28(5 ~ 48) months. One patient had a past medical history of hypertension and diabetes, 3 patients had hypertension. Symptoms induced by PVCs consisted of palpitations and chest pain. All patients in this study had failed prior antiarrhythmic therapy. The mean left ventricular ejection fraction (LVEF) was 64.88 ± 2.76 % (1 patients with a markedly decreased LVEF), and the left ventricular end-diastolic internal diameter (LVEDd) was 46.69 ± 2.28 mm (2 patients with a markedly dilated left ventricle). All patients were verified having no structural heart disease, including coronary artery disease, valvular heart disease, congenital heart disease, left ventricle hypertrophy, and right ventricle abnormalities by routine biochemistry tests, X-ray, color echocardiography examination, coronary angiography. Before RFCA, 12-lead ECGs of PVCs were obtained, 24 h ambulatory ECGs (Holter) were done at least once. The ECG was monitored for 24 h just before catheter ablation.

**Table 1:** Baseline patient characteristics

Patient	Age (y)	Sex	PVC count (number/24 h)	Symptoms	Symptom duration (M)	AADs used	Comorbidities	LVEF (%)	LVEDd (mm)
1	38	M	11026	palpitation	23	Beta-blocker, Propafenone	none	65	58
2	47	F	20348	palpitation	14	Beta-blocker	none	59	49
3	27	F	18265	palpitation	18	Beta-blocker	none	68	45
4	35	F	13529	palpitation	9	Beta-blocker, Mexiletine	none	67	44
5	24	M	22057	palpitation	22	Beta-blocker, Propafenone	none	66	47
6	41	M	32784	palpitation	38	Beta-blocker, Propafenone	none	69	48
7	21	F	12683	palpitation	16	Beta-blocker, Propafenone	none	66	46
8	27	F	21522	palpitation	32	Beta-blocker	none	63	49
9	15	M	32657	palpitation, chest pain	26	Beta-blocker, Amiodarone	none	62	43
10	62	M	32158	Palpitation chest pain	39	Beta-blocker, Mexiletine,	hypertension	66	41
11	34	F	26712	Palpitation	48	Beta-blocker	none	67	47
12	28	M	15336	Palpitation	18	Beta-blocker, Mexiletine	none	65	48
13	14	F	28637	Palpitation, chest pain	46	Beta-blocker, Propafenone	none	66	42
14	42	F	24512	Palpitation	13	Beta-blocker	none	59	47
15	13	M	24104	palpitation	16	Beta-blocker, Propafenone	none	65	51
16	22	F	16248	palpitation	10	Beta-blocker	none	58	46
17	65	F	16412	palpitation	16	Beta-blocker, Amiodarone	diabetes	62	47
18	37	M	20119	palpitation	6	Beta-blocker	none	62	46
19	29	M	SVT	palpitation	18	Beta-blocker	none	65	46
20	39	F	13008	palpitation, chest pain	6	Beta-blocker, Propafenone	none	62	48
21	25	M	20116	palpitation	26	Beta-blocker	none	65	49
22	36	M	16551	Palpitation	13	Beta-blocker, Propafenone	none	66	47
23	44	F	9807	palpitation, chest pain	28	Beta-blocker, Propafenone	none	68	47
24	58	M	16579	Palpitation	22	Beta-blocker, Propafenone	hypertension	66	49
25	33	F	22172	Palpitation	24	Beta-blocker, Propafenone	none	65	48
26	54	F	18613	Palpitation	20	-	hypertension	42	61
27	32	M	24007	palpitation	16	Beta-blocker, Amiodarone	none	65	46
28	27	F	21038	palpitation	5	Beta-blocker, Propafenone	none	63	47
29	29	M	15416	palpitation	22	Beta-blocker	none	67	48
30	32	F	19478	palpitation	24	Beta-blocker, Propafenone, Mexiletine	none	64	47
31	34	F	22342	palpitation	2	Beta-blocker, Propafenone,	none	68	46
32	33	F	12763	palpitation	6	Beta-blocker, Propafenone,	none	68	47
33	24	F	12913	palpitation	5	Beta-blocker, Propafenone,	none	70	46
34	22	F	15692	palpitation	24	Beta-blocker, Propafenone,	none	68	45
35	62	M	26314	palpitation	12	Beta-blocker	none	66	50
36	50	F	15213	palpitation	16	Beta-blocker, Propafenone,	none	65	48
37	24	F	16327	palpitation	38	Beta-blocker, Propafenone,	none	66	46
38	40	F	18706	palpitation	26	Beta-blocker, Propafenone,	none	66	48
39	50	M	20522	palpitation	32	Beta-blocker, Propafenone,	none	65	48
40	38	M	12637	palpitation	16	Beta-blocker, Propafenone,	none	68	46
Mean ± SD	33.53 ± 12.48		20231 ± 6102		20.08 ± 11.28			64.88 ± 2.76	46.69 ± 2.28

Annotation:*Y* years, *M* Months, *AADs* antiarrhythmic drugs, *LVEF* left ventricular ejection fraction, *LVEDd* left ventricular end-diastolic internal diameter

### Inclusion criteria

The selection criteria of patients for our study were as following: ① The QRS morphology of PVCs were qR pattern in at least one inferior leads with rightward axis, the QRS durations were between 0.08-0.13 s; ② The PVCs were monomorphic, with the average PVC counts ≥ 10000 times/24 h; ③ The PVCs caused clinical symptoms and refractory to at least one antiarrhythmic drugs, such as Beta-blocker, Amiodarone, Mexiletin, Propafenone; ④ No structural heart disease, and ⑤ Consent for the catheter ablation procedures.

### ECG measurements

The following ECG characteristics were assessed in patients with narrow PVCs: (1) The QRS morphology of the PVCs in inferior leads; (2) The precordial transitional zone; (3) The axis deviation; (4) The QRS complex duration. ECGs were reviewed by 2 investigators blinded to the site of origin; discrepancies were adjudicated by a third investigator. Capital letters (Q,R,S) refer to relatively high-amplitude waves (>5 mm). Conversely, lowercase letters (q,r,s) refer to relatively low-amplitude waves(≤5 mm).

### Eletrophysiologic study

Electrophysiologic study was performed after withdrawal of all anti-arrhythmic drugs for at least five half-lives. Eleven of 40 patients underwent the electrophysiologic study using three-dimensional mapping system (Ensite NavX system or Carto XP system or Carto 3 system), and 29 patients using conventional fluoroscopy guided mapping techniques. Six-French quadripolar catheters were introduced from the right femoral vein and placed across the tricuspid valve to record the His bundle (HB) activation and in the right ventricular apex for pacing. Mapping and pacing were performed using 7-F 4-mm-tip ablation catheters introduced from the right femoral vein (for the right ventricle) or right femoral artery (for the left ventricle). When few PVCs were observed at the beginning of the electrophysiologic study, induction of the PVCs was attempted by programmed electrical stimulation from the right ventricular apex with the addition of an isoproterenol infusion (2–4 μg/min). Programmed electrical stimulation was set at basic drive cycle lengths of 600, 500, and 430 msec, delivering a maximum of three extrastimulis.

### Mapping and radiofrequency catheter ablation

Activation and pace mapping were performed in all cases to identify the PVCs origin site. Pace mapping was performed at a pacing cycle length of 500 ms and stimulus amplitude of 1 mA greater than the late-diastolic threshold. When the earliest site of activation was mapped at a site near the aortic valve, angiography of the coronary artery and aortic root was performed before ablation to assess the anatomic relationships between those structures and the location of the ablation catheter. If the ablation site was close to the sinus of Valsalva, radiofrequency ablation was performed with an angiographic catheter positioned within the coronary artery ostium with frequent hand injections of contrast. Radiofrequency applications were delivered by temperature-controlled catheters with a target temperature of 55-60 °C and maximum power output of 30-40 W at sites exhibiting the earliest local ventricular activation with good pace match(pacing reproduced QRS morphology that is similar to the clinical PVC (≧11/12-lead concordance of major and minor deflections)). If the impedance is too high during ablation by temperature-controlled catheter, the irrigated-tip catheters were applied instead, with a target temperature of 43 °C and maximum power output of 30-35 W (with a 17 ml/min saline flow rate). When an acceleration or reduction of vemtricular tachycardia (VT) or PVCs was observed during the first 10s of radiofrequency application on target sites, the radiofrequency delivery was continued for 60 to 180 s. Otherwise, the radiofrequency delivery was terminated, and the ablation catheter was repositioned. When the earliest ventricular activation was observed with an HB potential or close to the HB region(an area within 5 mm from the site recording the largest HB potential) during the PVCs, radiofrequency energy was never delivered.

Programmed electrical stimulation and intravenous isoproterenol were repeated 30 min after the last application of radiofrequency to confirm the absence of PVCs before removing all catheters and sheaths.

### The definition of interleaflet triangle in aortic root

The aortic root spreading between the left ventricle and the tubular junction forms a central part of the heart. The aortic root consists of 3 sinuses of Valsalva, 2 of which (the right and left sinuses) make direct contact with the ostium of right and left coronary artery. The right (RCC) and left coronary cusps (LCC) incorporate ventricular musculature at their base but the non-coronary cusp (NCC) is exclusively composed of fibrous walls. There are three triangular shaped spaces in the aortic root between adjacent hinge lines of the cusps and the LV called interleaflet triangles. The interleaflet triangle between NCC and RCC relates to the membranous interventricular septum(IVS), has the central fibrous body located in, which contains the bundle of His. Hence, energy application in this site might be associated with a substantial risk for AV block. The interleaflet triangle between RCC and LCC relates to the muscular IVS and in close proximity to the site where left bundle branch bifurcates into two primary subdivisions, left anterior fascicle(LAF) and left posterior fascicle(LPF). The left interleaflet triangle between the NCC and LCC is an contiguous area from aortic-mitral curtain.

### Definition of the PVCs originating from the left anterior fascicle of left ventricular anterosuperior septum and L-RCC interleaflet triangle

PVC was considered originating from the LAF of left ventricular anterosuperior septum, based on (1) the local endocardial recordings; (2) the characteristic of left ventricular septum location (when viewed in the right and left anterior oblique fluoroscopic views after successful RFCA) and three-dimensional electroanatomical images; (3) Purkinje potentials were identified at successful sites during point mapping;(4) successful ablation achieved at Purkinje potential site.

PVC was considered originating from the L-RCC, based on (1) the local endocardial recordings; (2) in right anterior and left anterior oblique fluoroscopic view, angiography of the right and left coronary arteries and their related sinues of Valsava, showed the ablation catheter of target site was closely below the commissure between RCC and LCC, which was further confirmed by three-dimensional electroanatomical images; (3) Purkinje potentials were not identified at successful sites during point mapping; (4) unsuccessful ablation at Purkinje potential site.

### Post ablation follow-Up

After RFCA, all patients were not given any antiarrhythmic drugs and underwent 72-h ECG monitoring in hospital. Holters were followed 1 week, 1 month and 6 months after RFCA respectively at outpatient department. Transthoracic echocardiography was undergone 3 month after RFCA. ECG and 24-h ECG monitoring were performed whenever the patient had symptoms suggestive of recurrence of PVCs.

### Definition of successful ablation

Successful ablation was defined as (1) complete elimination of spontaneous PVCs after discharging on target sites, programmed electrical stimulation and intravenous isoproterenol 30 min after the last application of radiofrequency could not induce the PVCs, and (2) absence of the clinical PVCs on 72-h ECG monitoring post-ablation off antiarrhythmic drugs; (3) and no recurrence of the target PVCs in the absence of AADs during follow-up.

### Statistics analysis

Adopt Microsoft Office Excel 2003 statistics software, the measurement data were expressed by mean value ± standard deviation (χ ± s). The analysis of variance(ANOVA)and the student’s *t*-test were used in group comparison of measurement data. The numeration data were expressed by case number and percentage(%),the *χ*2-test or Fisher method were used to calculate probability.

### Ethics approval

Ethical approval was obtained from the Ethics Committee of the Second Affiliated Hospital of Wenzhou Medical University, and all participants consented to the experimental procedures. Written informed consent was obtained from each participant.

## Results

### Study population

A total of 40 patients were enrolled in the present study. According to the RFCA results, the 40 patients were divided into three groups: 19 in the left anterior fascicle group(LAF group), 16 in L-RCC interleaflet triangles group (L-RCC ILT group),5 in ablation failure group. The general clinic data of the three groups were showed in Table 2. Except that patients from L-RCC ILT had a longer duration of disease than patients from LAF (*p* < 0.01), there were no significant differences among the three groups with respect to age, PVCs counts per 24 h, parasystole case, hypertension cases, LVEF, LVEDd (*P* > 0.05).

**Table 2:** Comparsion of clinic data of the three groups

Group	Case	Age	Disease course/M	PVC times in 24 ambulatory electrocardiogram	Pararrhythmia case	Hypertesion case	LVEF(%)	LVEDd (mm)
L-RCC ILT	16	32.31 ± 12.93	25.07 ± 12.62*	22132 ± 7849	9	1	65.31 ± 2.66	46.69 ± 4.29
LAF	19	35.67. ± 13.20	16.00 ± 7.52	18391 ± 4702	12	1	64.02 ± 3.13	47.55 ± 1.34
Failure	5	34.80 ± 10.94	17.40 ± 7.54	19710 ± 3159	3	1	60.20 ± 10.28	49.80 ± 6.30

Annotation: compared with LAF, *p < 0.01. None patient have left ventricle dysfunction, *LVEF* left ventricular ejection fraction, *LVEDd* left ventricular end diastole internal diameter, *L-RCC ILT* Left coronary cusp-Right coronary cusp interleaflet triangle, *LAF* left anterior fascicle

### Results of electrophysiologic study, mapping and ablation

The PVCs occurred spontaneously in 32 patients and required electrical stimulation and isoproterenol infusion (2 ug) in 8 patients during the electrophysiologic study. Sustained ventricular tachycardia was not induced by electrical stimulation and isoproterenol infusion in all patients.

After systemic mapping, RFCA was then applied in 40 patients, total 35 patients got rid of PVCs after the procedure. Ablation at the site with the Purkinje potential recorded in left anterosuperior septum was effective in 19 patients, thus the origin of PVCs from left anterior fascicle was confirmed, the local ventricular activation time recorded at successful ablation sites preceded the onset of the QRS complex by 31.78 ± 3.27 ms, pace mapping of target sites showed a good match to spontaneous PVCs (concordance in 11.45 ± 0.65 leads). For another 16 patients, successful ablation was achieved at L-RCC interleaflet triangle where no Purkinje potentials were recorded, the local ventricular activation time recorded at target sites preceded the onset of the QRS complex by 24.92 ± 5.11 ms, pace mapping of target sites also showed a good match to spontaneous PVCs(concordance in 11.50 ± 0.71 leads). For 5 patients failed to the procrdure of RFCA, they had been mapped systemically in left anterosuperior septum and L-RCC interleaflet triangles and other anatomical structures of the heart, it was showed that the sites of earliest local ventricular activation time were in left ventricle close to the HB region and preceded the onset of the QRS complex by 18.8 ± 2.28 ms, pacing the target sites showed a poor pace match to spontaneous PVCs (concordance in 9.20 ± 0.45leads), no Purkinje potentials were recorded at target sites, radiofrequency energy was not delivered on the target sites for the fare of induction of inadvertent complete AV block.

The operations came off smoothly without ablation related complications. The results of RFCA are summarized in Table 3.

**Table 3:** Comparison of RFCA results

Group	Patient	The time of earliest ventricular activation preceding the QRS oneset(ms)	The similar lead counts between spontaneous PVCs and pace	Purkinje potential in target site	RF lesions prior to success	Operation time(min)	Radiation exposure time(min)	Ablation success rate(%)
L-RCC ILT	16	24.92 ± 5.11*	11.50 ± 0.71*	0	2.92 ± 1.50*	61.53 ± 11.29**	11/98 ± 5.17	16(100.00) *
LAF	19	31.33 ± 3.98*#	11.45 ± 0.65*	19*#	2.63 ± 1.09*	56.17 ± 13.80*	9.21 ± 3.96**	19(100.00) *
Failure	5	18.8 ± 2.28	9.20 ± 0.45	0	6.80 ± 0.83	79.16 ± 13.72	15.48 ± 3.69	0

Annotation: compared with ablation failure group, *p < 0.01, **p < 0.05;compared with L-RCC ILT, # *p* < 0.01, *L-RCC ILT* Left coronary cusp-Right coronary cusp interleaflet triangle, *LAF* left anterior fascicle

Patients have been followed-up for a mean period of 23 months without antiarrhythmic medications. No one patient had recurrent PVCs after RFCA.

### 12-lead ECG characteristics of PVCs from the three groups

**Fig. 1:**
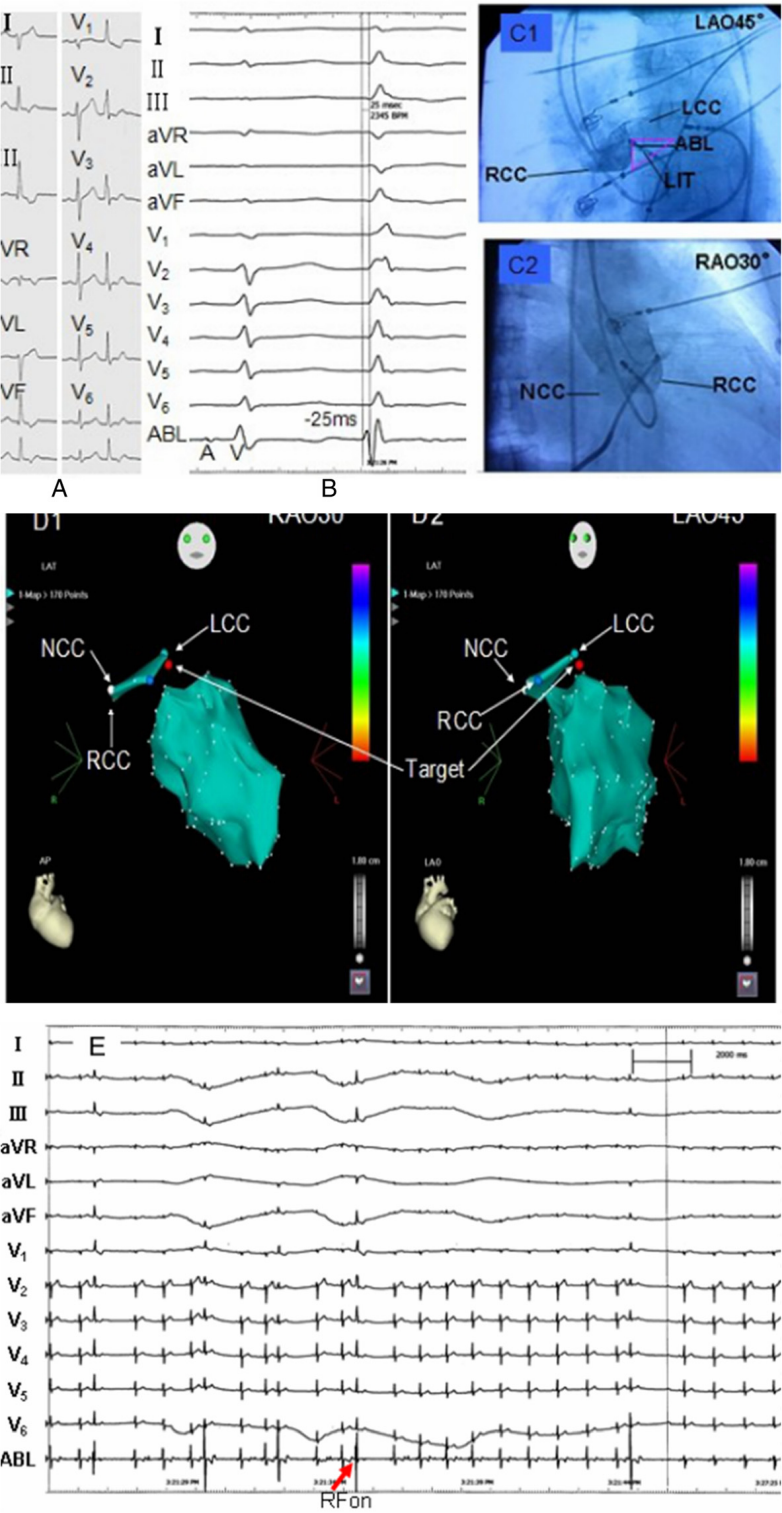
Mapping and ablation of PVCs from L-RCC LIT. **a**. 12-lead electrocardiogram of PVCs; **b**. RF energy had been applied on the site with earliest Purkinje potential in left ventricular anterosuperior septum, but the PVCs was not ablated. During PVCs, the earliest ventricular activation preceding the QRS onset by 25 ms was found in L-RCC interleaflet triangle and no Purkinje potential was recorded at this site. **c1**~**c2** and **d1**~**d2** The target site was confirmed by both angiography and three-dimension electroanatomical system. **e**. The PVCs diappeared within 10s during discharging on target site and the radiofrequency delivery was continued for 60 to 180 s. The patient has been followed up for 2.4 years without recurrence of PVCs. Annotation: PP=Purkinje potential, ABL=Ablation, RAO=Right anterior oblique position, LAO=Left anterior oblique position, RCC= Right coronary cusp, LCC=Left coronary cusp, NCC=noncoronary cusp, ILT=Interleaflet triangles, RFon=Radiofrequency on

**Fig. 2:**
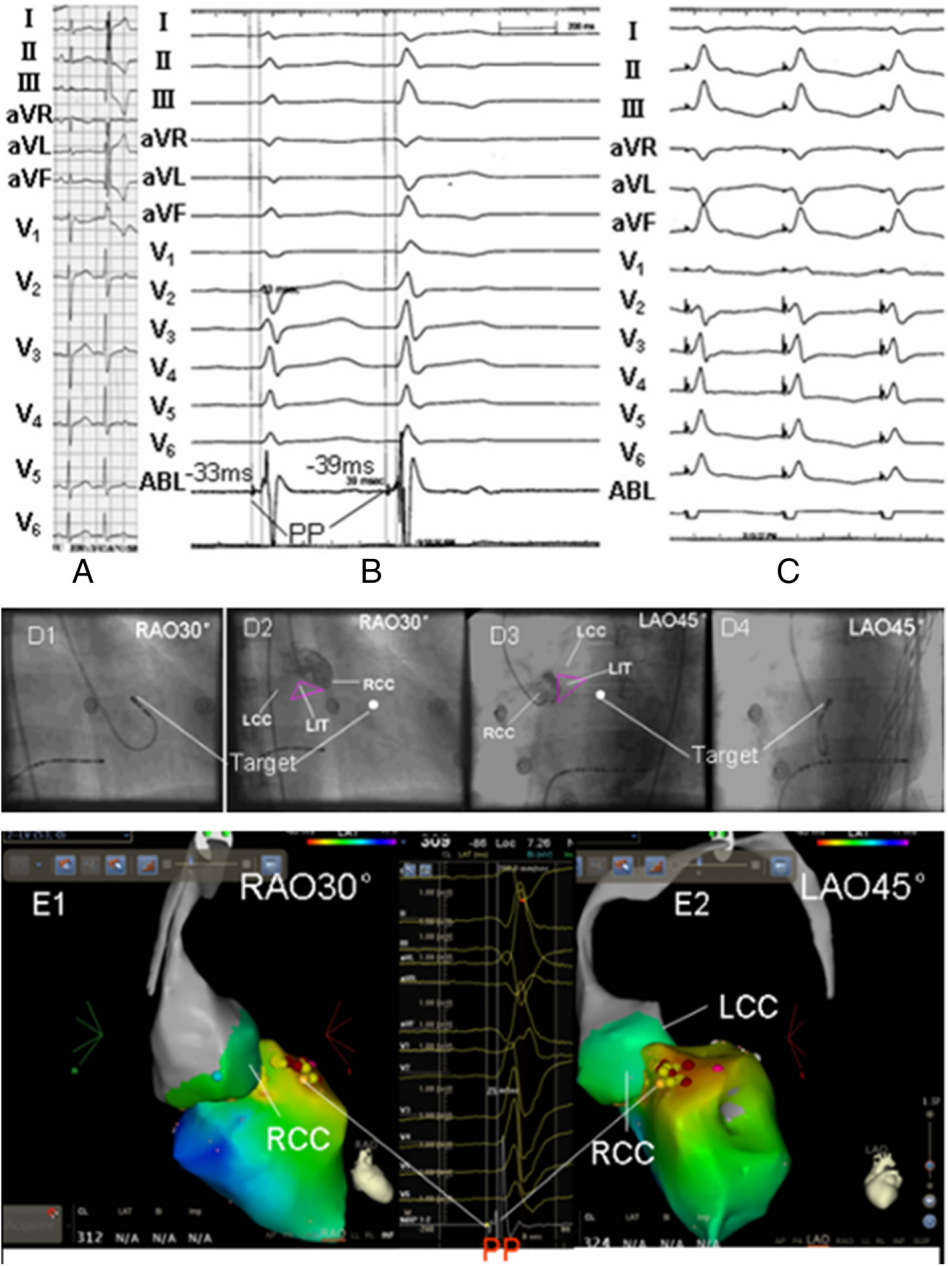
Mapping and ablation of PVCs from LAF in left anterosuperior septum. **a**.12-lead electrocardiogram of PVCs; **b**. The Purkinje potential mapped within left ventricular anterosuperior septum was 33 ms earlier the QRS onset in sinus rhythm and 39 ms earlier than the QRS onset in PVCs; **c**. Pacing the target sites of earliest Purkinje potential led to a perfect match, pacing reproduced QRS morphology was similar to the clinical PVCs in 11 leads; **d1**~**d4** and **e1**~**e2** The target site was comfirmed by X-ray image and three-dimension electroanatomical system. RF on target site led to successful ablation, no recurrence of PVCs in the follow-up of 1.4 years. Annotation: **e1**-**e2**, the red spots represented target site; the light green spots represented the Purkinje potentials

**Fig. 3:**
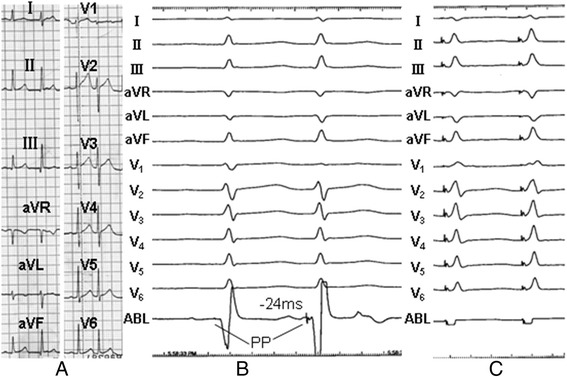
Mapping and ablation of PVCs from Failure group. **a**. 12-lead electrocardiogram of PVCs; **b**. The earliest Purkinje potential preceded the QRS onset by 22 ms in sinus rhythm and preceded the QRS onset by 24 ms in PVCs, RF on the site of earliest Purkinje potential did not terminate the PVCs; **c**. Pacing the sites mapped with earliest ventricular activation led to a poor match, pacing reproduced QRS morphology was similar to the clinical PVCs in just 9 leads of 12 leads, RF on the site of earliest ventricular activation did not terminate the PVCs

**Fig. 4:**
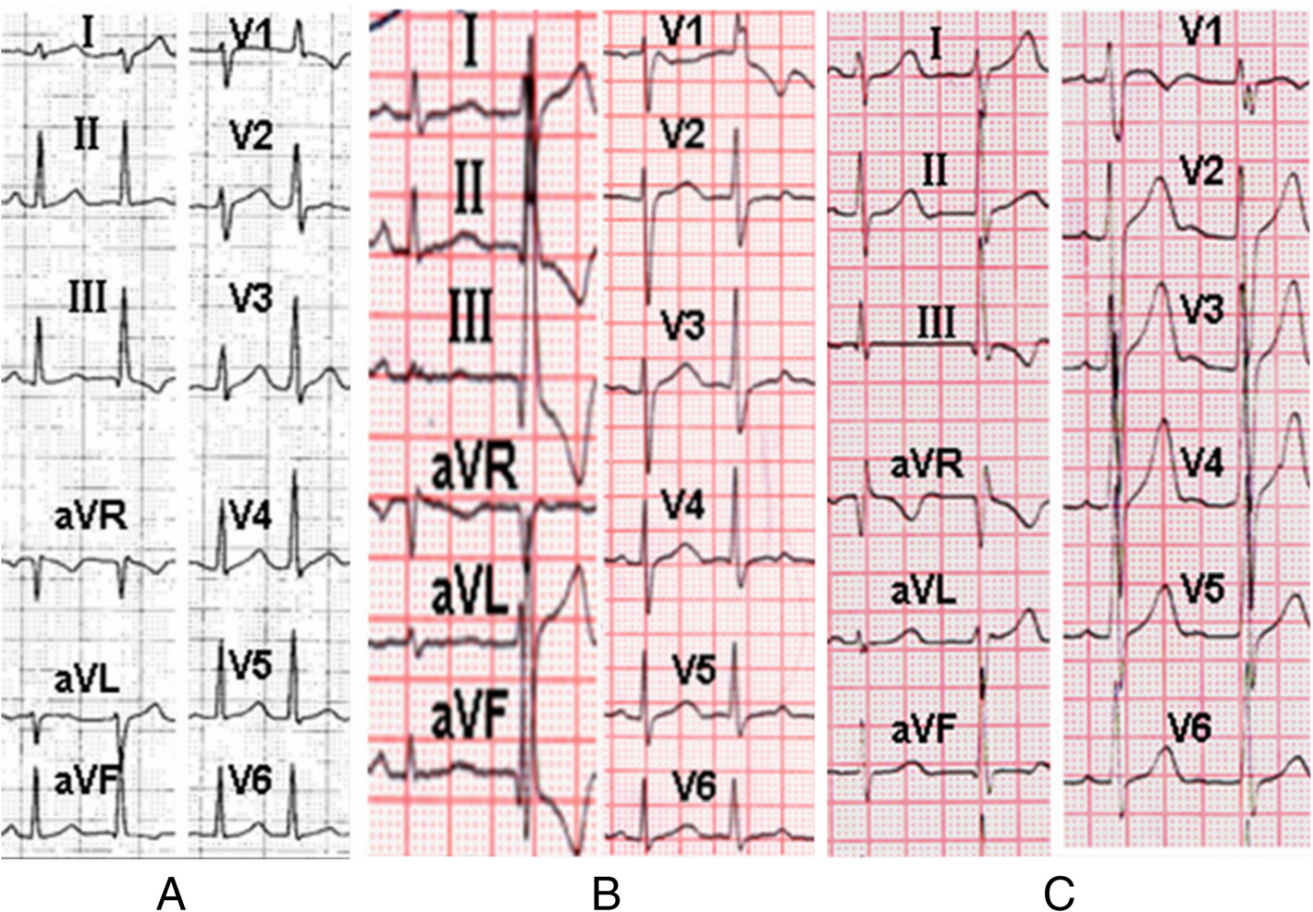
Representative 12-lead electrocardiogram of each group. **a** The 12-lead electrocardiogram PVCs originating from L-RCC ILT; **b** The 12-lead electrocardiogram of PVCs originating from LAF; **c**. The 12-lead electrocardiogram of ablation failure group

Based on the ablation results for narrow PVCs with qR pattern in inferior wall leads, we concluded the 12-lead PVCs electrocardiographic characteristics of the three groups ①PVCs originating from LAF groups, the QRS morphology were RBBB + LPFB with rightward axis, and the average QRS duration 116.07 ± 7.96 ms. The majority PVCs of this group had R or rsR’in lead V1, with their transition zone ahead of lead V1 in precordial leads. ②In PVCs originating from L-RCC ILT, the QRS complex were similar to PVCs originating from LAF in morphology but much narrower in QRS duration(100.44 ± 3.49 ms). They were also R or Rs in lead V1 with the transition zone was ahead of lead V1. ③In PVCs of ablation failure group, the QRS complex were similar to the former two groups in limb leads, but had the narrowest QRS duration (91.80 ± 6.94 ms). They were rs or rS (r/s or r/S≦1) in lead V1 with the precordial transition zone behind lead V3. The summarized ECG characterisitics of PVCs for each group were presented in Table 4. The ECG characterisitics of PVCs for each patient were displayed in Table 5. One representative case of PVCs ablation from each group was exhibited in Figs. 1, 2 and 3. One 12-lead electrocardiograms of PVCs from each group were presented in Fig. 4.

**Table 4:** QRS complex morphology of PVCs

Pt	Origin	QRS axis	QRS complex morphology												Transition zone	QRS duration (ms)
			V1	V2	V3	V4	V5	V6	I	aVR	aVL	II	III	aVF		
1	L-R ILT	right	Rs	Rs	Rs	Rs	Rs	Rs	rS	Qr	rS	qR	qR	qR	<V1	97
2	L-R ILT	right	R	Rs	Rs	Rs	Rs	Rs	rs	Qr	rS	qR	qR	qR	<V1	98
3	L-R ILT	right	Rs	RS	Rs	Rs	Rs	Rs	rS	Qr	rS	qR	qR	qR	<V1	100
4	L-R ILT	right	Rs	Rs	Rs	Rs	Rs	Rs	rS	Qr	rS	qR	qR	qR	<V1	106
5	L-R ILT	right	Rs	Rs	Rs	Rs	Rs	Rs	rS	Qr	rS	qR	qR	qR	<V1	97
6	L-R ILT	right	Rs	Rs	Rs	Rs	Rs	Rs	rS	Qr	rS	qR	qR	qR	<V1	102
7	L-R ILT	right	Rs	Rs	Rs	Rs	Rs	Rs	rs	Qr	rS	qR	qR	qR	<V1	103
8	L-R ILT	right	Rs	Rs	Rs	Rs	Rs	Rs	rS	Qr	rS	qR	qR	qR	<V1	96
9	L-R ILT	right	R	Rs	Rs	Rs	Rs	R	rS	Qr	rS	qR	qR	qR	<V1	101
10	L-R ILT	right	qRs	Rs	Rs	Rs	Rs	Rs	rS	Qr	rS	R	qR	qR	<V1	100
11	L-R ILT	right	R	Rs	Rs	Rs	Rs	R	rs	qr	rS	qR	qR	qR	<V1	98
12	L-R ILT	right	Rs	Rs	Rs	Rs	Rs	Rs	rS	Qr	rS	qR	qR	qR	<V1	101
13	L-R ILT	right	Rs	Rs	Rs	Rs	Rs	Rs	rS	Qr	rS	qRs	qR	qR	<V1	105
14	LAF	right	R	RS	Rs	Rs	Rs	R	rs	qr	rS	qR	qR	qR	<V1	116
15	LAF	right	R	RS	Rs	Rs	Rs	Rs	rS	Qr	rS	qR	qR	qR	<V1	112
16	LAF	right	R	RS	Rs	Rs	Rs	Rs	rs	Qr	rS	qR	qR	qR	<V1	118
17	LAF	right	R	Rs	Rs	Rs	Rs	Rs	rS	Qr	rS	qR	qR	qR	<V1	116
18	LAF	right	R	RS	RS	RS	Rs	Rs	rS	Qr	rS	qR	qR	qR	<V1	130
19	LAF	right	R	RS	RS	RS	Rs	Rs	rS	Qr	rS	qR	qR	qR	<V1	132
20	LAF	right	R	RS	RS	RS	Rs	Rs	rS	Qr	rS	qR	qR	qR	<V1	120
21	LAF	right	R	RS	RS	RS	Rs	Rs	rS	Qr	rS	qR	qR	qR	<V1	123
22	LAF	right	R	RS	RS	RS	Rs	Rs	rS	Qr	rS	qR	qR	qR	<V1	122
23	LAF	right	rsR’	RS	Rs	Rs	Rs	Rs	rs	QS	rS	qR	qR	qR	<V1	110
24	LAF	right	qR	Rs	Rs	Rs	Rs	Rs	rS	QS	rS	qR	qR	qR	<V1	123
25	LAF	normal	qR	RS	Rs	Rs	Rs	Rs	Rs	QS	RS	qR	qR	qR	<V1	101.3
26	Failure	right	rs	RS	RS	RS	Rs	R	rs	QS	rS	qR	qR	qR	V3	80
27	Failure	right	rs	RS	RS	RS	Rs	Rs	rS	Qr	rS	Rs	qRs	qRs	V3 ~ V4	98
28	Failure	right	rs	RS	RS	RS	Rs	Rs	rs	Qr	rS	qR	qR	qR	V3	95
29	Failure	right	rS	RS	RS	RS	Rs	Rs	rs	Qr	rS	qR	qR	qR	V3	94
30	Failure	right	rs	RS	RS	Rs	Rs	Rs	rs	Qr	rS	R	qR	qR	V3	92
31	L-R ILT	right	R	Rs	Rs	Rs	Rs	Rs	rs	Qr	rS	qR	qR	qR	<V1	97
32	LAF	right	rsR’	RS	RS	Rs	Rs	Rs	rs	QS	rS	qR	qR	qR	<V1	112
33	LAF	right	R	Rs	Rs	Rs	Rs	Rs	rs	QS	rS	qR	qR	qR	<V1	108
34	LAF	right	R	RS	RS	RS	Rs	Rs	rS	QS	rS	qR	qR	qR	<V1	106
35	L-R ILT	right	R	Rs	Rs	Rs	Rs	Rs	rs	Qr	rS	qR	qR	qR	<V1	101
36	LAF	right	R	Rs	Rs	Rs	Rs	Rs	rs	QS	rS	qR	qR	qR	<V1	108
37	LAF	right	qR	qR	qRs	qRs	Rs	Rs	rS	Qr	rS	qR	qR	qR	<V1	120
38	LAF	right	rsr’	Rs	Rs	Rs	Rs	qRs	Rs	QS	rS	qR	qR	qR	<V1	103
39	LAF	right	R	RS	RS	RS	Rs	Rs	rS	QS	rS	qR	qR	qR	<V1	125
40	L-R ILT	right	R	Rs	Rs	Rs	Rs	Rs	rs	QS	rS	R	qR	qR	<V1	106

Annotation: Capital letters (Q,R,S) refer to relatively high-amplitude (>5 mm). Conversely, lowercase letters (q,r,s) refer to relatively low-amplitude waves (<5 mm). *L-R ILT* Left coronary cusp-right coronary cusp interleaflet triangle, *LAF* left anterior fascicle

**Table 5:** Summaries of electrocardiogram characteristics of the three groups

Group	Number	QRS pattern in leads V1				QRS pattern in leads V2-V3			Transition Zone		QRS duration (ms)
		R or rsR’	Rs	rs or rS	qRs or qR	Rs orqRs	RS(R/S > 1)	RS(R/S ≤ 1)	≤V1	V3-V4	
L-RCC ILT	16	6	9*	0	1	14*	2	0	16*	0	100.44 ± 3.49**
LAF	19	16*^#^	0^#^	0	3	6^#^	12	1	19*	0	116.07 ± 7.96*^#^
Failure	5	0	0	5	0	0	0	5	0	5	91.80 ± 6.94

Annotation: compared with ablation failure group, *p < 0.01, **p < 0.05; compared with RCC-LCC LIT, # *p* < 0.01

*L-RCC ILT* Left coronary cusp-right coronary cusp interleaflet triangle, *LAF* left anterior fascicle

## Discussion

In the present study, a group of idiopathic PVCs, which presented narrow QRS complex (QRS duration < 130 msec) with qR pattern in inferior leads were enrolled to undergo the procedure of RFCA, most of the PVCs can be eliminated by energy delivery on the proximity of left anterior fascicle within left anterosuperior septum or L-RCC interleaflet triangles, for no significant complications related to RFCA happened and no documented recurrence was reported, we thought that RFAC was an effective and optimal therapy for this type of idiopathic PVCs.

It is confirmed that PVCs originating within the intraventricular conduction system are narrower than PVCs that occur outside the bundle branch system. This is because the bundle branch system supports the conduction in a different but relatively normal fashion. For the PVCs investigated in present study all presented narrow QRS morphology, they were thought arising from site within or closely near the intraventricular conduction system.

Previous study reported that the left anterior fascicular PVCs have an inferior, right ward direction, appearing positive in lead II, III, aVF, and negative in lead I, has an rSR’ configuration in precordial lead V1, since the impulse orignates within the left ventricular fascicles and the rigth bundle branch is last to be activated [14]. Of our findings in this study subjects, the electrocardiographic characteristics of the PVCs arising from the LAF were exactly consistent with the findings of the prior report. In PVCs originating from LAF in left ventricular anterosuperior septum,the QRS complex was RBBB + LPFB with an inferior, rightward axis. The majority PVCs of this group were R or rsR’in lead V1, with precordial transition zone before V1. Their QRS duration was 116.07 ± 7.96 ms. The origins of LAF were further confirmed by that these PVCs was successfully terminated by delivery of RF energy on the proximal portion of the LAF, where a spike presystolic purkinje potential preceding the ventricular potential during the PVCs and sinus rhythm was reproducibly observed. Based on our study, we thought that PVCs arising from LAF was one of the eminently ablatable ventricular arrhythmias and catheter ablation for this kind PVCs has a high success(100 %).

In the PVCs originating from L-RCC ILT,their electrocardiographic characteristics were similar to PVCs originating from LAF but much narrower in with QRS duration (100.44 ± 3.49 ms), they were R or Rs in lead V1, and the precordial transition zone was also before V1 lead. On the basis of the electrocardiographic characteristics of these PVCs, we initially considered that these PVCs were arising from LAF, however, RF delivery on the sites with presystolic Purkinje potentials recorded did not terminated the arrhythmias, thus the origins of PVCs from the left anterior fascicular Purkinje network area were excluded. After systemic mapping, the earliest local ventricular activation of PVCs were found at L-RCC interleaflet triangle without Purkinje potential recorded, and catheter ablation performed on this site completely excluded the arrhythmias. According to this phenomenon, we postulated these PVCs arising from L-RCC LIT may have preferential conduction pathway to the proximal portion of LAF, when the ventricular ectopic activity was triggered automatically in L-RCC LIT, the impulse conducted along the preferential pathway with breakout site at LAF, the involvement of LAF in the process of depolarization resulted in PVCs morphology similar to those PVCs arising from LAF. Recent studies may provide the rationale for our speculation. First, Yamada et al. has confirmed that L-RCC ILT can be a source of PVCs [15]. To the best of our knowledge, the aortic root consists of 3 sinuses of Valsalva, the attachment of the aortic valvular cusps results in 3 triangular extensions bound by thin fibrous walls of the aorta between the expanded sinuses, located approximately 1 cm above the base of either cusp, named interleaflet triangles. At the base of the LCC and RCC, the ventricular myocardium of the ostium of the left ventricle comes in direct contact with the aorta. The ventricular myocardium extending into the L-RCC ILT forming the ostium of the left ventricle at the same level as that connecting with the LCC and RCC, can be a potential trigger of ventricular arrhythmias. Second, recent studies showed it is sometimes difficult to precisely located the origin of ventricular arrhythmias in the aortic sinus cusp area because of the existence of preferential conduction pathway and associated anisotropic conduction [16]. Thirdly, anatomic studies have revealed a close relationship between the L-RCC ILT and the LAF. The anterior part of the RCC is adjacent to the bifurcating atrioventricular bundle and the origin of the left bundle branch. More anteriorly, the L-RCC ILT is related to the bifurcating left bundle branch and the most proximal part of LAF [17]. Above all, we thought that PVCs arising from L-RCC LIT having a similar electrocardiographic characteristics to those arising from LAF may be related to the preferential conduction pathway.

In ablation failure group, the QRS complex was similar to former two groups in limb leads but different in precordial leads. They were rs or rS(r/s or r/S < 1) in lead V1, the precordial transition zone was behind V3 lead, and they had the narrowest QRS duration (91.80 ± 6.94 ms). On the basis that the electrocardiographic characteristics of thses PVCs are very similar to the PVCs arising from the RV antero-septum just above the HB region once reported by Yuki Komatsu et al. [18], we thought that the origins of these PVCs are much closer to his bundle. Further systemic mapping performed by us showed that the sites of earliest local ventricular activation time of these PVCs were recorded in left ventricle, in close proximiry to the His bundle, radiofrequency energy was not delivered on the target sites for the fare of induction of inadvertent complete AV block.

## Conclusion

Most of idiopathic PVCs of narrow QRS duration (<130 msec) with qR pattern in inferior leads can be cured by the procedure of RFCA. Despite these narrow PVCs present similar QRS morphology, they are actually arising from different origins. When the ablation procedure failed at proximity of LAF within left anterosuperior septum, mapping and ablation in L-RCC ILT can be tried. The present findings can be helpful for planning catheter ablation for narrow PVCs manifesting qR in inferior leads.

### Study limitations

As only 40 patients of narrow PVCs were reviewed in our study,we still need observe more patients to further confirm our findings. In PVCs originating from L-RCC LIT group, the majority of target sites were verified by angiogranphy combined with three-dimension mapping system, however, additional intracardiac echocardiography was not obtained to further confirm the anatomical structure of origin in aortic root. Yamada et al. previously reported successful ablation of ventricular arrhythmias (VAs) at the junction between the LCC and RCC (L-RCC) in 5 cases, their eminent work confirmed that the structure of L-RCC LIT could be precisely localized by coronary angiography and three-dimension mapping system [15]. Consistent with the method described in Yamada’s work, in our study, when PVCs were considered arising from L-RCC LIT, selective angiography of the coronary artery and aorta have been performed before ablation to precisely define the location of the ablation catheter, the outline of the ASCs was observed by hand injections of contrast every 15 s. In the present study, the existence of preferential conduction pathway between L-RCC ILT and proximal portion of LAF was put forward to elucidate the similar QRS morphology of narrow PVCs from different origin, however, further validations were still required.

## Availability of data and materials

The datasets supporting the conclusions of the article are included within the article.

## Abbreviations

ECG: electrocardiogram
HB: His bundle
ILT: interleaflet triangle
IVS: interventricular septum
IVT: idiopathic ventricular tachycardia
LAF: left anterior fascicle
LCC: left coronary cusp
LPFB: left posterior fascicle block
LPF: left posterior fascicle
LVEDd: left ventricular end-diastolic internal diameter
LVEF: left ventricular ejection fraction
NCC: non coronary cusp
PVCs: premature ventricular contractions
RCC: right coronary cusp
RFCA: radiofrequency catheter ablation
VAs: ventricular arrhythmias
